# Lipocalin‐2 mediates the rejection of neural transplants

**DOI:** 10.1096/fj.202001018R

**Published:** 2021-01-09

**Authors:** Yi‐Chinn Weng, Yu‐Ting Huang, I‐Chen Chiang, Pei‐Ju Tsai, Yu‐Wen Su, Wen‐Hai Chou

**Affiliations:** ^1^ Center for Neuropsychiatric Research National Health Research Institutes Miaoli Taiwan; ^2^ Immunology Research Center National Health Research Institutes Miaoli Taiwan

**Keywords:** graft, Lipocalin‐2, neuroinflammation, neutrophil gelatinase‐associated lipocalin, rejection, transplantation

## Abstract

Lipocalin‐2 (LCN2) has been implicated in promoting apoptosis and neuroinflammation in neurological disorders; however, its role in neural transplantation remains unknown. In this study, we cultured and differentiated Lund human mesencephalic (LUHMES) cells into human dopaminergic‐like neurons and found that LCN2 mRNA was progressively induced in mouse brain after the intrastriatal transplantation of human dopaminergic‐like neurons. The induction of LCN2 protein was detected in a subset of astrocytes and neutrophils infiltrating the core of the engrafted sites, but not in neurons and microglia. LCN2‐immunoreactive astrocytes within the engrafted sites expressed lower levels of A1 and A2 astrocytic markers. Recruitment of microglia, neutrophils, and monocytes after transplantation was attenuated in LCN2 deficiency mice. The expression of M2 microglial markers was significantly elevated and survival of engrafted neurons was markedly improved after transplantation in LCN2 deficiency mice. Brain type organic cation transporter (BOCT), the cell surface receptor for LCN2, was induced in dopaminergic‐like neurons after differentiation, and treatment with recombinant LCN2 protein directly induced apoptosis in dopaminergic‐like neurons in a dose‐dependent manner. Our results, therefore, suggested that LCN2 is a neurotoxic factor for the engrafted neurons and a modulator of neuroinflammation. LCN2 inhibition may be useful in reducing rejection after neural transplantation.

**Abbreviations**
DAPI: 4′, 6‐diamidino‐2‐phenylindole
GAPDH: glyceraldehyde‐3‐phosphate dehydrogenase
GFAP: glial fibrillary acidic protein
Iba1: ionized calcium‐binding adapter molecule 1
MAP2: microtubule‐associated protein 2
MTT: 2; 3‐(4,5‐dimethylthiazol‐2‐yl)‐2,5‐diphenyltetrazolium bromide
PFA: paraformaldehyde
TUNEL: terminal transferase‐mediated dUTP nick end labeling

## 1 INTRODUCTION

Cell replacement therapy has been investigated as a potential treatment for neurological disorders including Parkinson's disease (PD), traumatic brain injury, and ischemic stroke.^1^, ^2^ Intracerebral transplantation may, however, provoke an immune response that results in the rejection of the engrafted neurons.^3^ Long‐term systemic administration of immunosuppressive agents is required to ensure the survival of grafts; however, immunosuppression may lead to severe side‐effects, such as nephrotoxicity, hypertension, neurotoxicity, diabetes, and considerable morbidity.^4^, ^5^ Thus, understanding the immune mechanisms of graft rejection is essential to improve cell replacement therapy for neurological disorders.

Studies have indicated that within a few hours after grafting, hypoxia and anoikis cause cell death in the core of engrafted neurons due to the absence of blood vessels in the cellular grafts.^6^, ^7^ Phagocytic neutrophils, monocytes, and macrophages are then recruited to the graft site, in response to the apoptosis and necrosis of hypoxic transplanted cells.^3^ Next, the graft is infiltrated and surrounded by activated microglia and astrocytes. Pro‐inflammatory chemokines and cytokines enhance the phagocytic activity and free‐radical production of microglia and peripheral immune cells, resulting in direct cell‐death of implanted neurons. Reactive astrocytes form an effective barrier to separate the graft from surrounding brain tissue.^8^ There has been evidence of the infiltration of astrocytes in neural grafts, although their roles remain elusive.^9^, ^10^ Astrocytes are a heterogeneous group of glial cells that provide trophic factors for neurons, control the formation and maintenance of synapses, and regulate the release and uptake of neurotransmitters.^11^, ^12^, ^13^, ^14^, ^15^, ^16^ In response to brain injuries and diseases, astrocytes undergo morphological and biochemical changes called “reactive astrocytosis”. Two different types of reactive astrocytes (A1 and A2) have been described.^12^, ^16^ A1 astrocytes lose the ability to promote neuronal survival, outgrowth, and synaptogenesis, and are potentially detrimental to neurons and oligodendrocytes.^12^, ^16^ Moreover, A2 astrocytes with the upregulation of several neurotrophic factors and are proposed to be neuroprotective.

Lipocalin‐2 (LCN2), also known as 24p3 or neutrophil gelatinase‐associated lipocalin (NGAL), is a 25‐kDa protein that is acutely produced and secreted from neutrophils, astrocytes, endothelial cells, and microglia in response to infection, inflammation, and injury.^17^ Previous studies have demonstrated the ability of LCN2 to induce apoptosis through intracellular iron sequestration.^18^, ^19^ LCN2 is internalized by its cell surface receptor, namely brain type organic cation transporter (BOCT), chelates intracellular iron, and then exits the cell, resulting in a net iron loss and apoptosis.^19^ Based on this mechanism, we have investigated the potential roles of LCN2 and BOCT in neural transplantation. In this study, we report the upregulation of LCN2 in reactive astrocytes infiltrating the core of the graft and BOCT expression in the engrafted neurons. The immunoreactivities of A1 (C3) and A2 (S100A10) astrocytic markers were lower in LCN2‐immunoreactive astrocytes. Numbers of infiltrating microglia and immune cells were reduced, and microglia displayed the alternatively activated phenotype (M2) in LCN2 deficiency mice after transplantation. The survival of engrafted neurons was improved significantly in LCN2 deficiency mice. In vitro treatments with recombinant LCN2 resulted in apoptosis of the engrafted neurons. These results provide support for roles of LCN2 and BOCT in the rejection of engrafted neurons and promotion of neuroinflammation after transplantation, suggesting the inhibition of LCN2‐BOCT signaling as a novel therapeutic mechanism to reduce the rejection of neural transplants.

## 2 MATERIALS AND METHODS

### 2.1 LUHMES cell culture and differentiation

Lund human mesencephalic (LUHMES) cells are derived from 8‐week‐old human ventral mesencephalon, immortalized by a tetracycline‐regulated v‐myc‐vector, and can be differentiated into morphologically and biochemically mature dopaminergic‐like neurons with a high conversion rate (>99%).^20^, ^21^ The LUHMES cells were obtained from ATCC (Manassas, VA, USA) and cultured following a previously published procedure with minor modifications.^20^, ^21^ For the two‐dimensional (2D) LUHMES culture,^20^ cells were seeded in plastic culture flasks coated with poly‐l‐ornithine (50 μg/mL, Sigma‐Aldrich, St. Louis, Missouri, USA) and human fibronectin (1 μg/mL, Sigma‐Aldrich), and allowed to proliferate in the Advanced DMEM/F‐12 medium (Thermo Fisher Scientific, Waltham, MA, USA) containing 1x N‐2 supplement (Thermo), 1x GlutaMAX (Thermo), and 40 ng/mL of recombinant basic fibroblast growth factor (bFGF, Thermo) in a humidified incubator with 5% CO_2_ at 37°C. After 3 to 4 days of proliferation, the LUHMES cells were differentiated for 6 days following the 2‐step procedure with 1 mM dibutyryl‐cAMP (Sigma‐Aldrich), 1 μg/mL of doxycycline (Sigma‐Aldrich), and 2 ng/mL of recombinant human glial cell‐derived neurotrophic factor (GDNF, Thermo).^20^

Since three‐dimensional (3D) culture represents closer cell‐to‐cell interactions and reproduces better in vivo physiology,^21^ the LUHMES cells were trypsinized after 3 to 4 days of proliferation, placed in plastic culture plates on a gyratory shaker at 80 rpm in a humidified incubator with 10% CO_2_ at 37°C, and differentiated for 4 to 5 days with 1 mM dibutyryl cAMP (Sigma‐Aldrich), 1 μg/mL of doxycycline (Sigma‐Aldrich), and 2 ng/mL of recombinant human GDNF (Thermo).

### 2.2 Bright‐field imaging and immunocytochemistry

Bright‐field images of 2D and 3D LUHMES cells were collected using a Canon EOS 650D camera mounted onto a Leica DM IL inverted microscope. For immunofluorescence staining, 2D and 3D LUHMES cells were fixed with 4% PFA in PBS and incubated overnight at 4°C with anti‐MAP2 (1:200, Millipore, Burlington, MA, USA), anti‐tyrosine hydroxylase (TH, 1:200, Abcam, Cambridge, UK), and anti‐BOCT (1:200, ProSci, Poway, CA, USA) antibodies.^22^, ^23^ After washing, the cells were stained with Alexa Fluor conjugated secondary antibodies (1:200, Jackson ImmunoResearch, West Grove, PA, USA) at room temperature for 1 hour and mounted in media containing DAPI (Vector Laboratories, Burlingame, CA, USA). The images were collected using a Leica TCS SP5 II confocal laser scanning microscope.

### 2.3 Cell transplantation

All animal experiments were conducted in accordance with the Institutional Animal Care and Use Committee policies at National Health Research Institutes. Male *LCN2^+/+^* and *LCN2* ^−/−^ mice on a C57BL/6 background (Jackson Laboratory, Bar Harbor, ME, USA) between 2 and 4 months of age were anesthetized with isoflurane (induction at 4% and maintained at 1% inhalation), and positioned in a stereotaxic frame (Stoelting, Wood Dale, IL, USA). During anesthesia, body temperature was monitored and maintained at 37.0°C using a Homeothermic Blanket System (Fine Scientific Tools, Foster City, CA, USA), with a needle Thermistor probe (ThermoWorks, American Fork, UT, USA). After the midline scalp incision, a small hole was drilled through the skull. 3D LUHMES neurospheres after 4 to 5 days of differentiation (~2.75 × 10^5^ in 10 μL) were loaded into a 10 μL gastight Hamilton syringe and 26G Hamilton needle, and injected at a rate of 0.5 μL/min over 20 minutes into the right striatum (0.5 mm AP, −2.0 mm ML from bregma; −3.5 mm DV from dura) using an Ultra MicroPump III (World Precision Instruments, Sarasota, FL, USA).^24^, ^25^ The syringe and needle were left in place for 5 more minutes after transplantation. The drilled hole was sealed with bone wax and the incision was sutured. Mice were returned to their home cages on a heating pad until fully recovered from anesthesia.

### 2.4 Immunohistochemistry

Mice were anesthetized with 4% isoflurane in N_2_O/O_2_ (70%/30%) at different time points after transplantation and perfused transcardially with 4% PFA in 0.15 M phosphate buffer (pH 7.3).^23^, ^26^ Coronal sections (30 μm) of fixed brains were prepared using a Leica CM3050 S Cryostat and incubated overnight at 4°C with anti‐MAP2 (1:200, Abcam), anti‐human nuclei specific marker (HuNu) (1:200, Millipore), anti‐LCN2 (1:200, R&D), anti‐Iba1 (1:200, Wako, Osaka, Japan), anti‐GFAP (1:1000, DAKO, Glostrup, Denmark), anti‐Ly‐6B.2 clone 7/4 (1:200, Abcam), anti‐C3 (1:200, Abcam) or anti‐S100A10 (1:200, Abcam) antibodies. After washing, the sections were stained with Alexa Fluor conjugated secondary antibodies (1:200, Jackson ImmunoResearch), and mounted in media containing DAPI (Vector). The images were acquired using a Leica TCS SP5 II confocal microscope. Numbers of HuNu‐immunoreactive neurons in the striatal graft regions (−2.0 mm caudal to bregma) were counted by an investigator blinded to the genotypes. LCN2‐, C3‐, and S100A10‐immunoreactive astrocytes were outlined using the Freehand selection tool in NIH ImageJ. Twenty astrocytes per mouse (n = 3) were selected for the quantification of LCN2 vs C3, or LCN2 vs S100A10 immunoreactivity. The immunoreactivities of LCN2, C3, and S100A100 in astrocytes as well as the sizes (µm^2^) of LCN2‐, C3‐, and S100A10‐immunoreactive astrocytes were measured. The percentage of LCN2, C3, and S100A100 immunoreactivity was calculated using the following formulas.

$$\text{LCN2}\;\left(\%\right)=\text{LCN2}/\left(\text{LCN2}+\text{C3 or S1}00\text{A1}0\right)\;\times 100.$$

$$\text{C3}\;\left(\%\right)\;\text{or S1}00\text{A1}0\;\left(\%\right)=\text{C3 or S1}00\text{A1}0/\left(\text{LCN2}+\text{C3 or S1}00\text{A1}0\right)\;\times 100.$$

### 2.5 Real‐time RT‐PCR

Mice were anesthetized with 4% isoflurane in N_2_O/O_2_ (70%/30%) at different time points after transplantation and perfused transcardially with 0.15 M phosphate buffer (pH 7.3). Total RNA was isolated from the ipsilateral (right) hemisphere using TRIzol reagent (Invitrogen, Carlsbad, CA, USA) and MagNA Lyser Green Beads in a MagNA Lyser Instrument (Roche, Indianapolis, IN, USA). Total RNA was isolated from LUHMES cells using the RNeasy Plus Mini Kit (Qiagen, Hilden, Germany). RNA was quantified by NanoDrop 2000 Spectrophotometer (Thermo) and reverse transcribed into cDNA using a RevertAid H Minus First Strand cDNA Synthesis Kit (Thermo) and a Veriti 96‐Well Thermal Cycler (Thermo). Real‐time PCR was performed using the StepOnePlus Real‐Time PCR System (Thermo) and Luminaris Color HiGreen High ROX qPCR Master Mix (Thermo). The mRNA levels of targeted genes were determined using the 2^−ΔΔCT^ method using mouse GAPDH mRNA as an internal control. The sequences of the primers are listed in Table 1.^27^, ^28^

**TABLE 1:** Nucleotide sequences of the primers used in real‐time RT‐PCR

Gene name	Primer sequences
*Mouse LCN2*	F, 5′‐ATG TCA CCT CCA TCC TGG TC‐3′
	R, 5′‐CAC ACT CAC CAC CCA TTC AG‐3′
*Mouse GFAP*	F, 5′‐AGG CAG AAG CTC CAA GAT GA‐3′
	R, 5′‐TGT GAG GTC TGC AAA CTT GG‐3′
*Mouse Iba1*	F, 5′‐GAA GCG AAT GCT GGA GAA A‐3′
	R, 5′‐GAC CAG TTG GCC TCT TGT GT‐3′
*Mouse iNOS*	F, 5′‐GCC ACC AAC AAT GGC AAC A‐3′
	R, 5′‐CGT ACC GGA TGA GCT GTG AAT T‐3′
*Mouse CXCL10*	F, 5′‐AAG TGC TGC CGT CAT TTT CT‐3′
	R, 5′‐GTG GCA ATG ATC TCA ACA CG‐3′
*Mouse TNFα*	F, 5′‐ATG GCC TCC CTC TCA TCA GTT C‐3′
	R, 5′‐TTG GTG GTT TGC TAC GAC GTG‐3′
*Mouse CCL2*	F, 5′‐TCA GCC AGA TGC AGT TAA CG‐3′
	R, 5′‐GAT CCT CTT GTA GCT CTC CAG C‐3′
*Mouse Arg1*	F, 5′‐CGC CTT TCT CAA AAG GAC AG‐3′
	R, 5′‐CCA GCT CTT CAT TGG CTT TC‐3′
*Mouse YM1*	F, 5′‐GGG CAT ACC TTT ATC CTG AG‐3′
	R, 5′‐CCA CTG AAG TCA TCC ATG TC‐3′
*Mouse CCL22*	F, 5′‐ATG GTG CCA ATG TGG AAG ACA‐3′
	R, 5′‐GGC AGG ATT TTG AGG TCC AGA‐3′
*Mouse GAPDH*	F, 5′‐CCA TTT GCA GTG GCA AAG‐3′
	R, 5′‐CAC CCC ATT TGA TGT TAG TG‐3′
*Human LCN2*	F, 5′‐GAA GTG TGA CTA CTG GAT CAG GA‐3′
	F, 5′‐ACC ACT CGG ACG AGG TAA CT‐3′
*Human BOCT*	F, 5′‐AAT CCT TAG AGA CAA GGG CCA‐3′
	F, 5′‐CCT GGG TGT CTA CCT GAT GC‐3′
*Human GAPDH*	F, 5′‐CAC CAT CTT CCA GGA GCG AGA TC‐3′
	F, 5′‐GCA GGA GGC ATT GCT GAT GAT C‐3′

Abbreviations: F, forward primer; R, reverse primer.

### 2.6 Flow cytometry

Mice were anesthetized with 4% isoflurane in N_2_O/O_2_ (70%/30%) at 1 and 7 days after transplantation and perfused transcardially with 0.15 M phosphate buffer (pH 7.3). Ipsilateral hemispheres were isolated and minced using the plunger end of a 5 mL syringe and a 100 μm cell strainer.^29^ Tissue suspensions were incubated with 1 mL of digestion buffer containing Liberase (2 U/mL, Roche) at 37°C for 1 hour, and dissociated into single‐cell suspensions through a 70 μm cell strainer. Myelin and cell debris were removed from cell suspensions by density gradient centrifugation. Cells were labeled with anti‐murine CD16/CD32 (eBioscience) to block Fc receptors, LIVE/DEAD Fixable Dead Cell Stain (Invitrogen), CD45.2‐FITC (eBioscience), Ly6G‐PerCP‐Cy5.5 (BD Bioscience), CD3‐PE (BioLegend), and CD11b‐Horizon V500 (BD), and CD19‐PE‐Cy7 (BioLegend). Acquisition and data analysis were performed using Attune NxT flow cytometer (Thermo Fisher Scientific) and FlowJo software (Tree Star, Inc, USA).

### 2.7 MTT assay

The cell viability of LUHMES neurons after treatments with recombinant human LCN2 protein (R&D) was determined by the Vybrant MTT Cell Proliferation Assay Kit (Thermo).^23^ 2D LUHMES neurons were incubated with 1.1 mM of MTT in a humidified CO_2_ incubator at 37°C for 4 hours. Twenty‐five μL of media was mixed with 50 μL of DMSO and incubated at 37°C for 10 minutes. The absorbance of the mixture was measured at 540 nm.

### 2.8 TUNEL assay and immunocytochemistry for detecting apoptotic neurons

Apoptosis in LUHMES neurons after treatments with recombinant human LCN2 protein (R&D) was detected by TUNEL assays using In Situ Cell Death Detection Kit, Fluorescein (Roche) according to the manufacturer's instructions. Immunofluorescence staining using anti‐cleaved Caspase‐3 (Asp175) (1:200, Cell Signaling) was used to detect apoptotic neurons after the treatments. After the staining, the number of apoptotic neurons labeled positively with TUNEL or cleaved Caspase‐3 was counted by an investigator blinded to the treatments using a 40x objective and a Leica TCS SP5 II confocal laser scanning microscope. The numbers of apoptotic neurons per mm^2^ were statistically analyzed between different groups.

### 2.9 Statistical analysis

Quantitative data were presented as means ± SEM and analyzed by *t*‐tests, one‐way ANOVA, and Newman‐Keuls post hoc tests using Prism 5 (GraphPad, La Jolla, CA, USA). The *F* statistics, degrees of freedom (*df*), and the overall *P* value for the pairwise comparisons are listed in Table S1. The *P* value less than .05 was considered to be statistically significant.

## 3 RESULTS

### 3.1 Characterization of human dopaminergic‐like neurons

**FIGURE 1:**
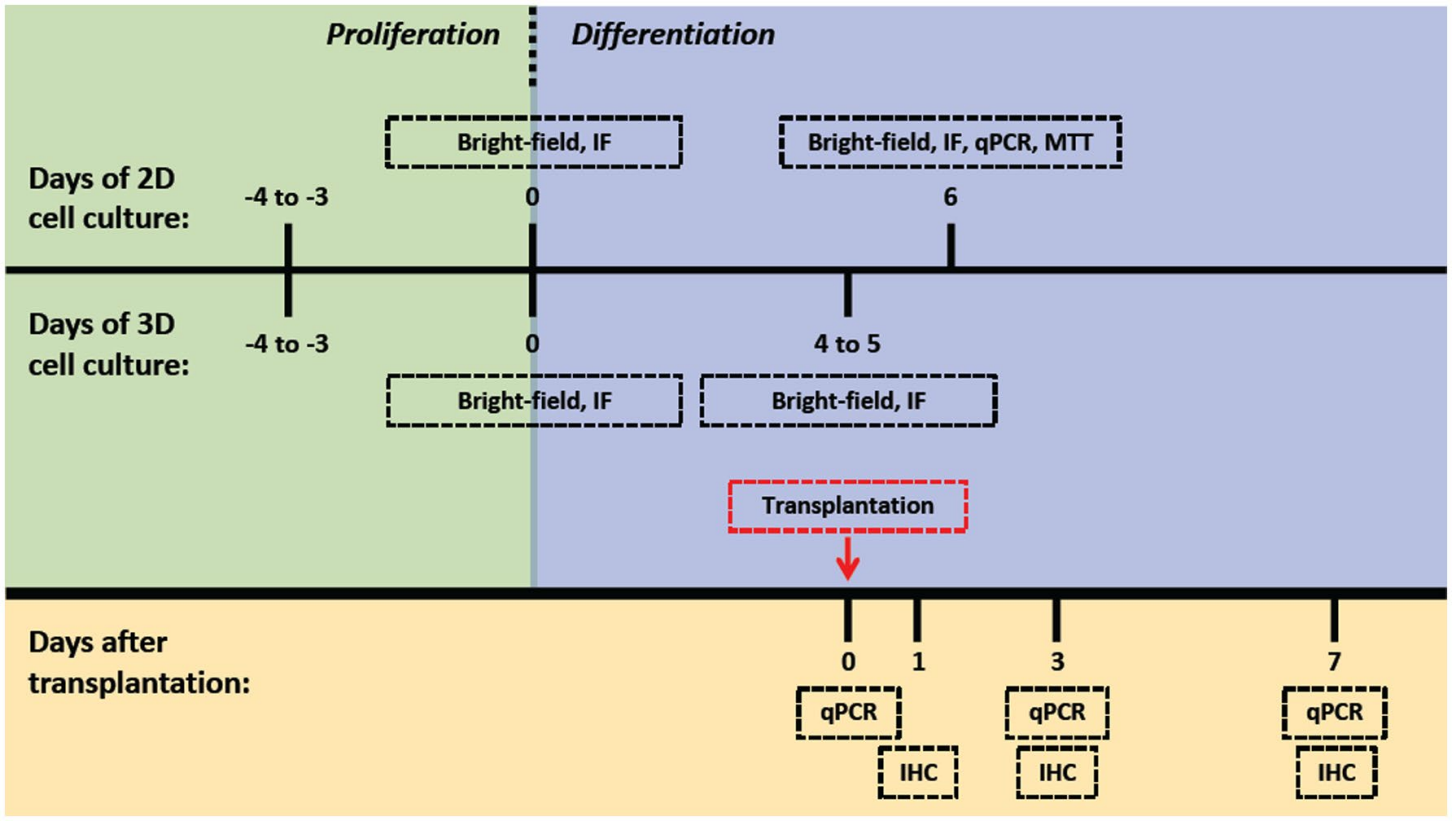
Experimental procedure. LUHMES cells were seeded and allowed to proliferate for 3 to 4 days before differentiation. Bright‐field and immunofluorescence (IF) images of 2D and 3D LUHMES cells were collected before and after differentiation. After 4 to 5 days of differentiation, 3D LUHMES neurospheres were transplanted into mouse brains. Mouse brains isolated before and after transplantation were analyzed by quantitative PCR (qPCR) and immunohistochemistry (IHC)

To obtain dopaminergic‐like neurons for transplantation, Lund human mesencephalic (LUHMES) cells were cultured and differentiated following a previously published procedure (Figure 1).^20^, ^21^ LUHMES neurons in both two‐dimensional (2D) cultures and three‐dimensional (3D) neurospheres formed an elaborate neurite network when switched to differentiation medium, and expressed both MAP2 and the dopaminergic neuron markers, tyrosine hydroxylase (TH) (Figures S1 and S2).

### 3.2 Induction of *LCN2* mRNA in mouse brain after transplantation

**FIGURE 2:**
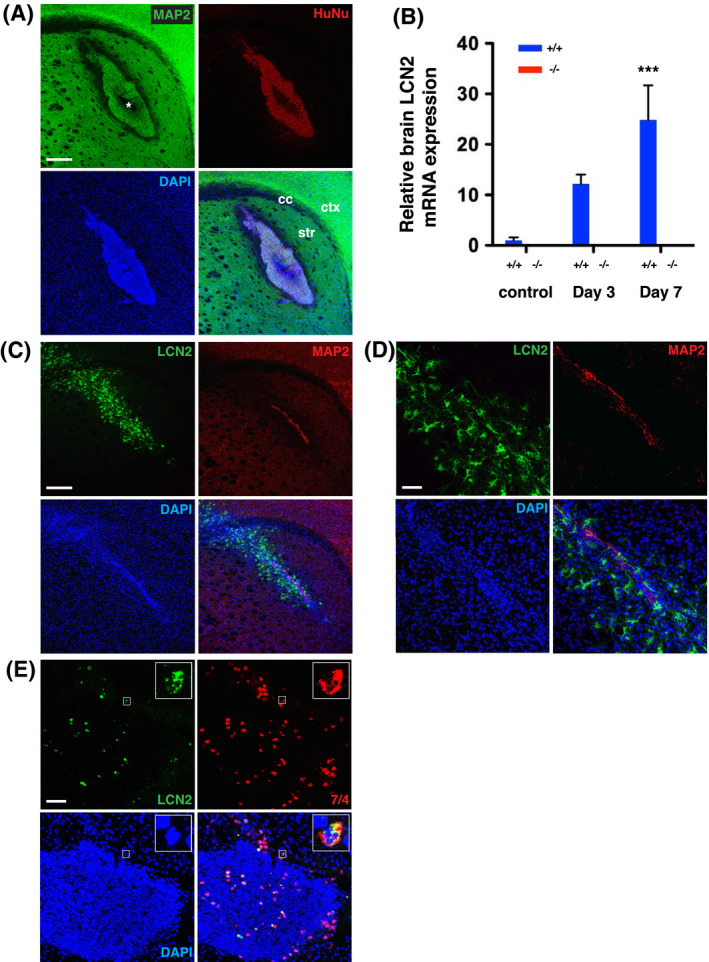
Induction of LCN2 after the transplantation of LUHMES neurospheres. A, Expression of MAP2 (green) and a human nuclei specific marker (HuNu, red) in the striatum was detected by immunofluorescence staining and confocal microscopy on day 1 after transplantation. Nuclei were labeled with DAPI (blue). HuNu‐immunoreactive LUHMES neurons were identified as a mass of cells in the striatum. * indicates the area of cell death induced by hypoxia and anoikis; cc, corpus callosum; ctx, cortex; str, striatum. B, Total RNA isolated from the ipsilateral hemispheres of naïve *LCN2^+/+^* and *LCN2* ^−/−^ mice (control), and 3 and 7 days after transplantation were analyzed by real‐time RT‐PCR (n = 4 per group). Relative expression *LCN2* mRNA in the brain homogenates was compared between groups using one‐way ANOVA and Newman‐Keuls post hoc tests. *LCN2* mRNA level was significantly induced 7 days after transplantation (***P < .001) as compared to naïve *LCN2^+/+^* mice. C and D, Immunoreactivity of LCN2 (green) and MAP2 (red) in the striatum on day 7 after transplantation. D, Higher magnification of confocal images demonstrating LCN2‐immunoreactive cells (green) induced around the MAP2‐immunoreactive LUHMES neurons (red). E. Immunoreactivity of LCN2 (green) and 7/4 (red) in the engrafted site in the striatum on day 1 after transplantation. Enlarged views of boxed areas showing that LCN2 immunoreactivity (green) in the cytoplasm of an infiltrating neutrophil with a polymorphonuclear nucleus (red). Scale bars, 250 μm in A and C, and 50 μm in D and E

To assess the role of LCN2 in neural transplantation, 3D LUHMES neurospheres were stereotaxically injected into the striatum of immune‐competent C57BL/6 mice (Figures 1 and 2). On day 1 after transplantation, LUHMES neurons displaying MAP2 and HuNu staining were visible as a mass of cells in the striatum (Figure 2A). To determine the expression of LCN2 in response to transplantation, ipsilateral hemispheres from *LCN2^+/+^* and *LCN2* ^−/−^ mice were isolated before, as well as 3 and 7 days after, transplantation and analyzed by real‐time RT‐PCR. Although the levels of *LCN2* mRNA were low in naive *LCN2^+/+^* mice and absent in *LCN2* ^−/−^ mice, they were induced progressively after transplantation (Figure 2B). Significant induction of *LCN2* mRNA was detected on day 7 after transplantation as compared to that in naive *LCN2^+/+^* mice.

### 3.3 Induction of LCN2 protein in a subset of neutrophils and reactive astrocytes infiltrating the core of engrafted sites

We next assessed the cellular localization of LCN2 after transplantation. Brain sections were stained with antibodies recognizing LCN2 and specific markers for neurons (MAP2), neutrophils (7/4), microglia (Iba1), or astrocytes (GFAP). Most engrafted neurons were eliminated 7 days after transplantation, while LCN2 protein was detected around the graft in the striatum (Figure 2C). Confocal images at higher magnification showed that LCN2 was not expressed in MAP2‐immunoreactive neurons after transplantation (Figure 2D). LCN2 protein was detected in 7/4‐immunoreactive neutrophils infiltrating the graft on day 1 (Figure 2E), but not on days 3, 5, or 7 after transplantation (Figure S3).

**FIGURE 3:**
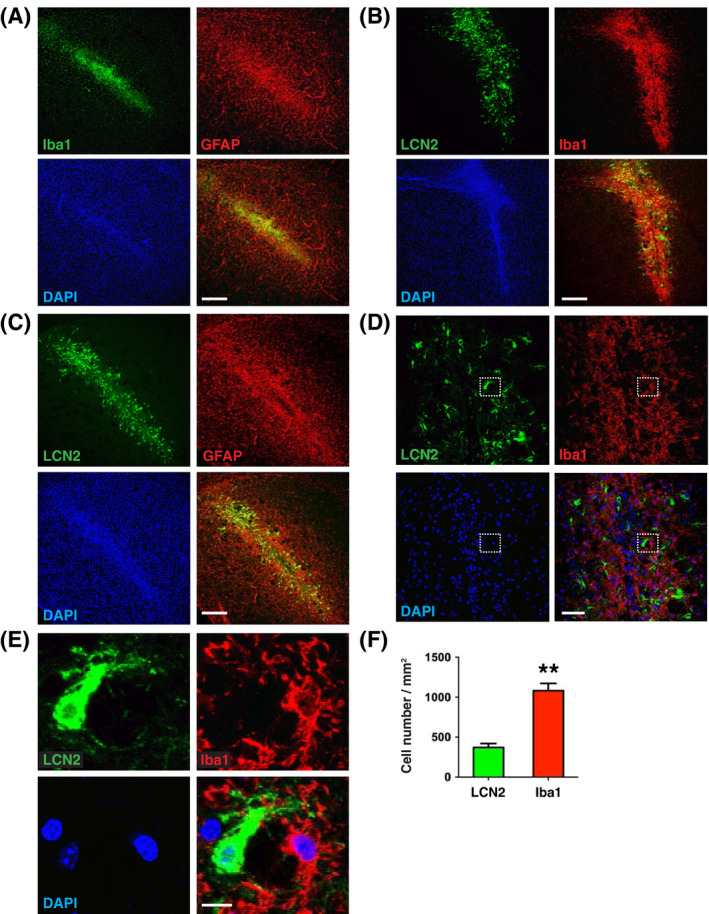
Spatial relationship between LCN2 expression, astrocytes, and microglia surrounding the graft. A, Recruitment of Iba1‐immunoreactive microglia (green) and GFAP‐immunoreactive astrocytes (red) in the striatum 7 days after transplantation. Induction of LCN2 (green) and Iba1 (red) (B, D) or GFAP (red) (C) in the striatum 7 days after transplantation. E, Enlarged views of the boxed area in D showing that LCN2 (green) is not expressed in the Iba1‐immunoreactive microglia (red). Nuclei were labeled with DAPI (blue). Scale bars, 250 μm in A‐C, 50 μm in D, and 5 μm in E. F, The numbers of LCN2‐ or Iba1‐immunoreactive cells on day 7 after transplantation (n = 3). **P < .001 compared between groups using the two‐tailed, unpaired *t*‐test

**FIGURE 4:**
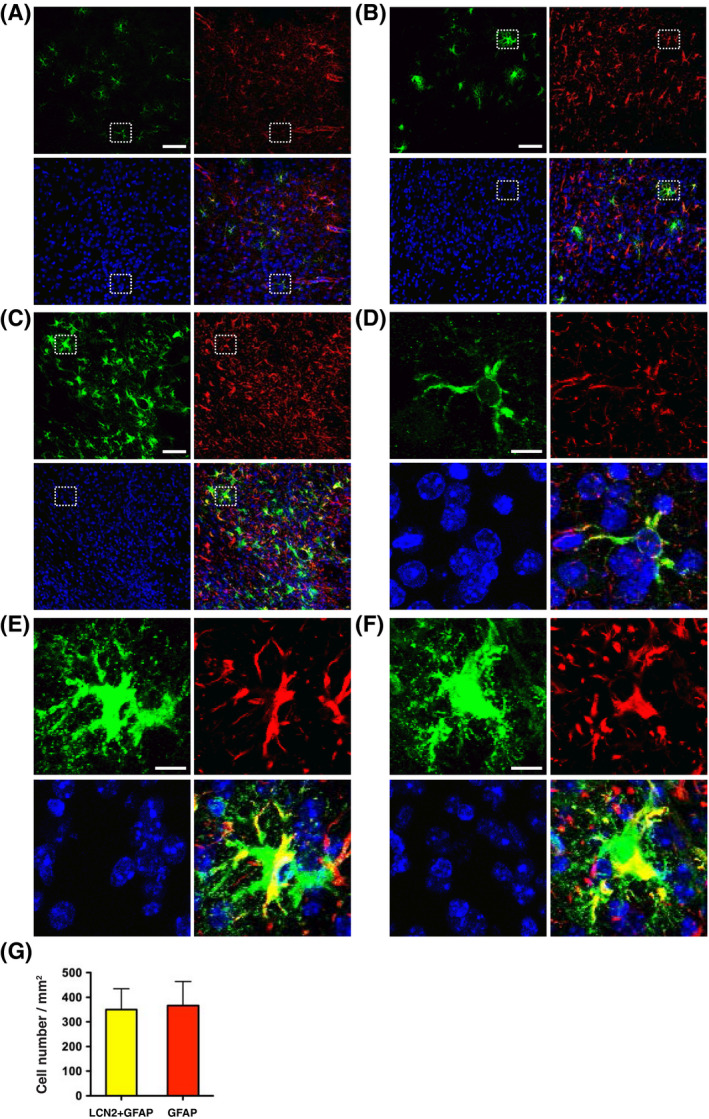
Detection of LCN2 in a subset of astrocytes after transplantation. Immunoreactivity of LCN2 (green) and astrocyte‐specific marker GFAP (red) in the striatum on day 1 (A), day 3 (B), and day 7 (C) after transplantation. D‐F, Enlarged views of boxed areas in A‐C showing the expression of LCN2 (green) in the GFAP‐immunoreactive astrocytes (red). Nuclei were labeled with DAPI (blue). Scale bars, 50 μm for the main images in A‐C, and 5 μm for the amplified images in D‐F. G, The numbers of astrocytes immunoreactive for both LCN2 and GFAP, or for GFAP only, on day 7 after transplantation (n = 3). *P* = .9058 compared between groups using the two‐tailed, unpaired *t*‐test

The engrafted neurons elicited a strong immune cell response and became highly surrounded and invaded by Iba1‐immunoreactive microglia and GFAP‐immunoreactive astrocytes within 7 days after transplantation (Figure 3A). Activated microglia were recruited to the core of the engrafted site, while the activated astrocytes were broadly distributed around the graft. LCN2‐immunoreactive cells were detected within the zone of activated microglia (Figure 3B) and located centrally in the midst of activated astrocytes (Figure 3C). LCN2 expression was not detected in Iba1‐immunoreactive microglia (Figure 3D,E), but rather, in a subset of GFAP‐immunoreactive astrocytes (Figure 4). The number of LCN2‐immunoreactive astrocytes increased gradually from day 1 to day 7 after transplantation (Figure 4), correlating with the progressive induction of *LCN2* mRNA (Figure 2B). LCN2‐immunoreactive astrocytes displayed morphological characteristics of reactive astrocytes at 3 and 7 days after transplantation, with hypertrophy of cell bodies and main processes (Figure 4E,F).^30^ The numbers of LCN2‐, Iba1‐, and GFAP‐immunoreactive cells in the striatum 7 days after transplantation were counted; there were 369.4 ± 49.44 (cells/mm^2^) LCN2‐immunoreactive cells, 1082 ± 91.48 (cells/mm^2^) Iba1‐immunoreactive microglia (Figure 3F), 350.4 ± 84.68 (cells/mm^2^; 48.9%) LCN2‐ and GFAP‐ immunoreactive astrocytes, and 366.7 ± 97.95 (cells/mm^2^; 51.1%) GFAP‐immunoreactive astrocytes (Figure 4G).

### 3.4 LCN2‐immunoreactive astrocytes in the engrafted sites expressed lower levels of C3 and S100A10 proteins

**FIGURE 5:**
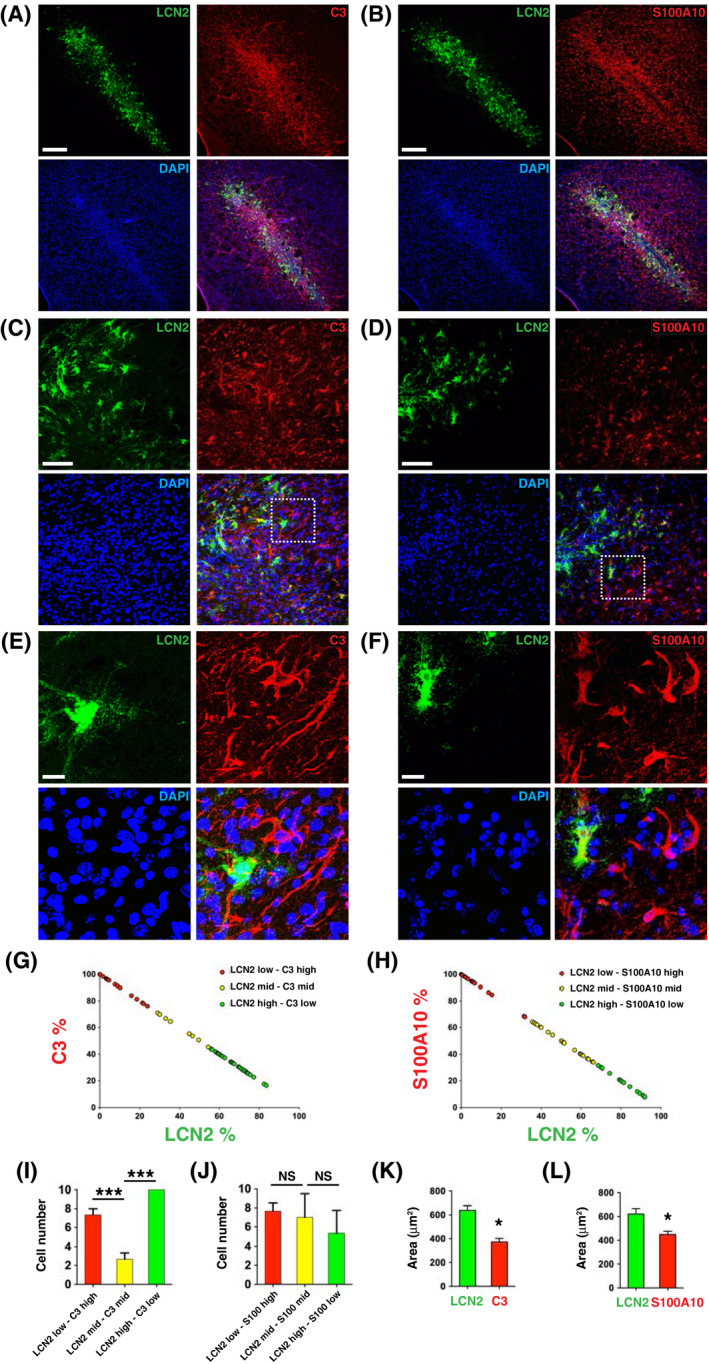
Immunoreactivity of LCN2 (green) and C3 (red) (A, C, E), or LCN2 (green) and S100A10 (red) (B, D, F) in the striatum on day 7 after transplantation. C and D, Higher magnification of confocal images demonstrating that LCN2‐immunoreactive astrocytes (green) were located closer to the engrafted site on the left. E and F, Enlarged views of boxed areas in C and D. Nuclei were labeled with DAPI (blue). Scale bars, 250 μm in A and B, 50 μm in C and D, and 5 μm in E and F. Percentages of LCN2 and C3 immunoreactivity (G), or LCN2 and S100A10 immunoreactivity (H) in astrocytes in the engrafted sites on day 7 after transplantation (n = 60). I, J, The numbers of astrocytes expressing a low level of LCN2 and a high level of C3 or S100A10 (red), medium levels of LCN2 and C3 or LCN2 and S100A10 (yellow), and a high level of LCN2 and a low level of C3 or S100A10 (green) (n = 3 per group). Cell numbers were compared between groups using one‐way ANOVA and Newman–Keuls post hoc tests (***P < .001). The areas (µm^2^) occupied by LCN2‐ and C3‐immunoreactive (K) or LCN2‐ and S100A10‐immunoreactive (L) astrocytes (n = 30 per group). *P < .05 compared with the area occupied by LCN2‐immunoreactive astrocytes (two‐tailed, unpaired *t*‐test)

Recent studies have demonstrated that there are at least two different types of reactive astrocytes (A1 and A2).^12^, ^16^ To assess whether LCN2 is expressed in A1 or A2 astrocytes, brain sections isolated 7 days after neural transplantation were stained with antibodies recognizing LCN2 and specific markers for A1 (C3) or A2 (S100A10) astrocytes. Interestingly, LCN2‐immunoreactive astrocytes were detected centrally in the engrafted site, while C3‐ and S100A10‐immunoreactive astrocytes were broadly distributed around the graft (Figure 5A,B). LCN2‐immunoreactive astrocytes within the engrafted site expressed lower C3 and S100A10 immunoreactivity, and displayed morphological hypertrophy with more processes (Figure 5C‐F).^31^ Moreover, C3‐ and S100A10‐immunoreactive astrocytes at the border of the engrafted site expressed lower LCN2 immunoreactivity, and displayed morphological atrophy with fewer processes. These astrocytes were divided into three groups based on the percentage of LCN2 immunoreactivity (low, medium, and high) (Figure 5G,H). There were significantly fewer astrocytes expressing medium levels of LCN2 and C3 compared to the number of those expressing mostly LCN2 or C3 (Figure 5I). The number of astrocytes expressing medium levels of LCN2 and S100A10 was similar to the number of cells expressing mostly LCN2 or S100A10 (Figure 5J). Moreover, the size of LCN2‐immunoreactive astrocytes was significantly larger than that of the C3‐ and S100A10‐immunoreactive astrocytes (Figure 5K,L).

### 3.5 Recruitment of microglia, neutrophils, and monocytes after transplantation was reduced in LCN2 deficiency mice

**FIGURE 6:**
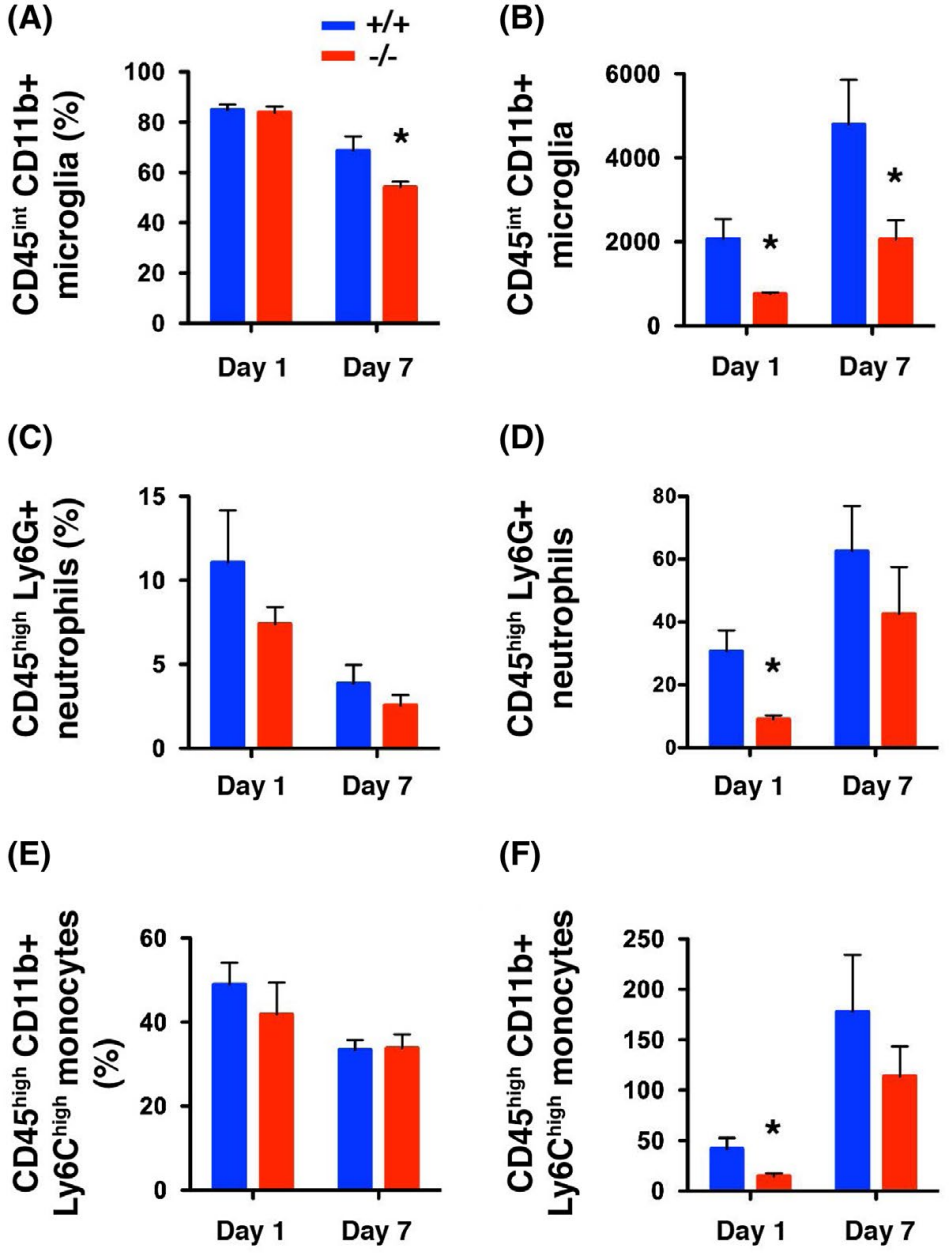
Recruitment of microglia, neutrophils, and monocytes after transplantation was attenuated in LCN2 deficiency mice. Flow cytometric analysis of the percentages (A, C, E) and numbers (B, D, F) of CD45^int^ CD11b+ microglia (A, B), CD45^high^ Ly6G+ neutrophils (C, D), and CD45^high^ CD11b+ Ly6C^high^ monocytes (E, F) in the ipsilateral hemisphere on day 1 and day 7 after transplantation (n = 5 per group). The percentages and numbers of microglia and immune cells were compared between *LCN2^+/+^* and *LCN2* ^−/−^ mice, on day 1 or day 7 after transplantation, using the two‐tailed, unpaired *t*‐test (*P < .05)

LCN2 has been implicated in recruiting resident microglia and peripheral immune cells after brain injury.^23^, ^32^, ^33^ To assess the infiltration of microglia and immune cells after transplantation, ipsilateral hemispheres of *LCN2^+/+^* and *LCN2* ^−/−^ mice were isolated on days 1 and 7 after transplantation and analyzed by flow cytometry (Figure S4). The percentage of CD45^int^ CD11b+ microglia was significantly attenuated 7 days after transplantation in *LCN2* ^−/−^ mice (Figure 6A) and the number of CD45^int^ CD11b+ microglia was significantly attenuated at 1 and 7 days after transplantation in *LCN2* ^−/−^ mice (Figure 6B). Although the percentages of infiltrating immune cells were similar in *LCN2^+/+^* and *LCN2* ^−/−^ mice (Figure 6 and Figure S5), the numbers of infiltrating CD45^high^ Ly6G+ neutrophils and CD45^high^ CD11b+ Ly6C^high^ monocytes were significantly reduced 1 day after transplantation in *LCN2* ^−/−^ mice (Figure 6). The percentages and numbers of infiltrating CD45^high^ CD3+ T cells, CD45^high^ CD19+ B cells, and CD45^high^ CD11b+ Ly6C^low^ monocytes/cDC/macrophages after transplantation were not significantly different between *LCN2^+/+^* and *LCN2* ^−/−^ mice (Figure S5). These results suggest that LCN2 mediates the recruitment of resident microglia, peripheral neutrophils, and monocytes after transplantation.

### 3.6 The expression of M2 microglial markers was elevated on day 7 after transplantation in LCN2 deficiency mice

**FIGURE 7:**
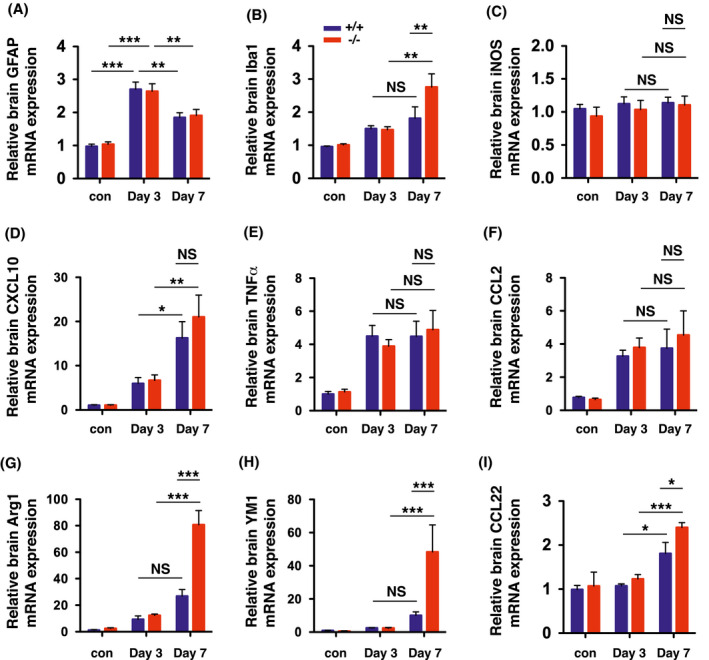
The expression of M2 microglial markers were elevated on day 7 after transplantation in LCN2 deficiency mice. Total RNA isolated from the ipsilateral hemispheres of naive *LCN2^+/+^* and *LCN2* ^−/−^ mice (control), and at 3 and 7 days after transplantation, were analyzed by real‐time RT‐PCR (n = 4 per group). Relative mRNA expression of the target genes in the brain homogenates was compared between groups using one‐way ANOVA and Newman–Keuls post hoc tests (***P < .001, **P < .01, *P < .05; NS, not significant)

LCN2 has been shown to modulate the phenotype polarization of astrocytes, microglia, and macrophages in response to inflammatory stimuli.^32^ To assess the role of LCN2 in the phenotypic polarization after neural transplantation, ipsilateral hemispheres of *LCN2^+/+^* and *LCN2* ^−/−^ mice were isolated before, and 3 and 7 days after, transplantation and analyzed by real‐time RT‐PCR (Figure 7). The expression of the astrocytic marker *GFAP* was significantly induced on day 3 in both *LCN2^+/+^* and *LCN2* ^−/−^ mice after transplantation compared to naïve mice of the same genotype and then reduced on day 7 compared to expression on day 3 (Figure 7A). No significant difference in *GFAP* expression was detected between *LCN2^+/+^* and *LCN2* ^−/−^ mice before or after transplantation. Interestingly, expression of the microglial marker Iba1 was significantly elevated in *LCN2* ^−/−^ mice compared to *LCN2^+/+^* mice at 7 days after transplantation (Figure 7B). While the expression of M1 microglial markers (iNOS, CXCL10, TNFα, CCL2) was similar between *LCN2^+/+^* and *LCN2* ^−/−^ mice (Figure 7C‐F), the expression of M2 microglial markers (Arg1, YM1, CCL22) was significantly elevated in *LCN2* ^−/−^ mice 7 days after transplantation (Figure 7G‐I). These results suggest that LCN2 plays an essential role in the suppression of the M2 polarization of microglia on day 7 after transplantation.

### 3.7 Improved survival of engrafted neurons in LCN2 deficiency mice

**FIGURE 8:**
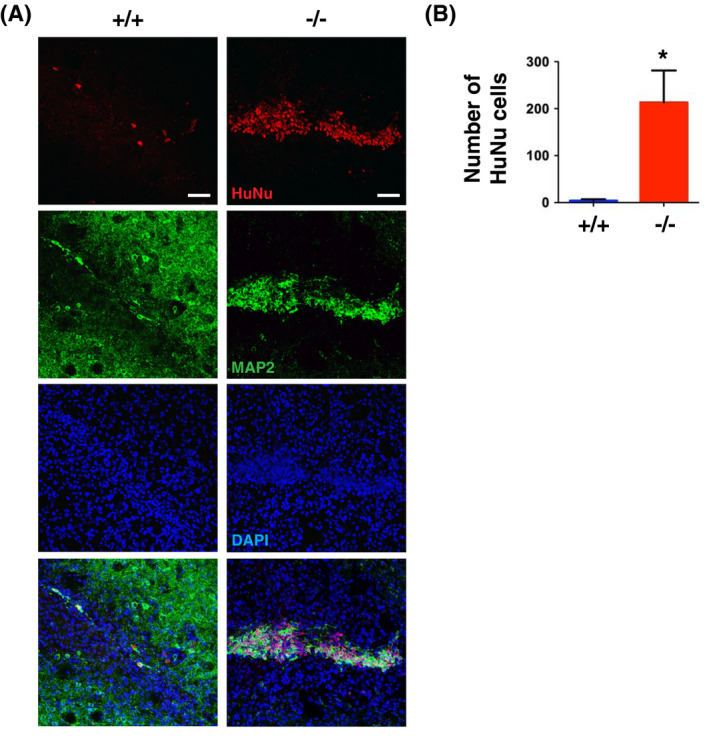
The number of surviving LUHMES cells after transplantation was increased in LCN2 deficiency mice. A, Confocal images of HuNu (red) and MAP2 (green) immunoreactivity in the striatum of *LCN2^+/+^* and *LCN2* ^−/−^ mice 7 days after transplantation. Scale bar, 50 μm. B, Significantly more HuNu‐immunoreactive cells were observed in *LCN2* ^−/−^ vs *LCN2^+/+^* mice (n = 5). *P < .05 compared with *LCN2^+/+^* mice (two‐tailed, unpaired *t*‐test)

To assess the function of LCN2 induced after transplantation, 3D LUHMES neurospheres were intrastriatally implanted in *LCN2^+/+^* and *LCN2* ^−/−^ mice. Confocal images showed that most of the engrafted neurons, stained positively with HuNu and MAP2 antibodies, were cleared out in the striatum of *LCN2^+/+^*, while many engrafted neurons survived in *LCN2* ^−/−^ mice (Figure 8A). On day 7 after transplantation, the number of HuNu‐immunoreactive neurons was significantly greater in the striatum of *LCN2* ^−/−^ than in that of *LCN2^+/+^* mice (Figure 8B). Our results showed that the survival of engrafted neurons was improved in the absence of LCN2, thus suggesting that LCN2 is essential in mediating the rejection of engrafted neurons.

### 3.8 Induction of apoptosis in differentiated LUHMES neurons by recombinant LCN2

**FIGURE 9:**
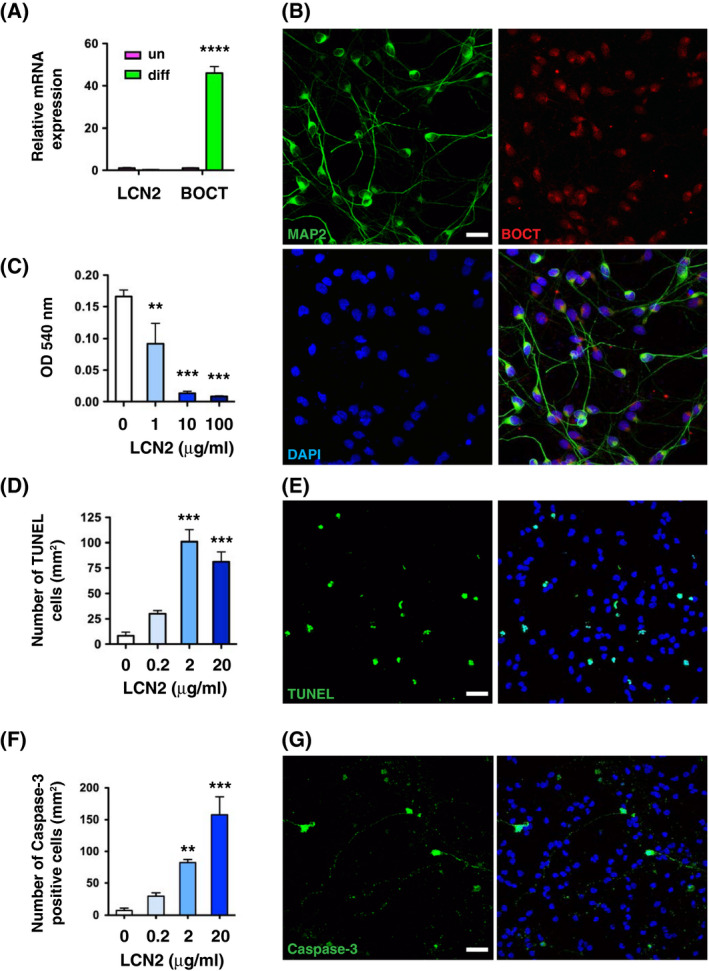
LCN2 induced cell death in 2D LUHMES neurons. A, Total RNA was isolated from undifferentiated (un) 2D LUHMES cells and from cells 6 days after differentiation (diff). RNA was analyzed by real‐time RT‐PCR (n = 5). Relative expression of *LCN2* and *BOCT* mRNA was compared between undifferentiated and differentiated cells using the two‐tailed, unpaired *t*‐test. BOCT mRNA expression was significantly increased at 6 days after differentiation compared to undifferentiated cells (****P < .0001). B, Confocal images showing the expression pattern of BOCT in 2D LUHMES neurons. Neurons were stained for the neuronal marker MAP2 (green) and BOCT (red). C‐G, 2D LUHMES neurons were incubated with increasing concentrations of recombinant human LCN2 protein at 37°C for 24 hours. C, The viability of neurons after LCN2 treatment was determined by MTT assays (n = 5). D, The number of apoptotic neurons labeled by TUNEL assay after LCN2 treatment (n = 6). E, Confocal images showing the TUNEL‐positive neurons (green) after treatment with recombinant human LCN2 protein (2 μg/mL). F, The numbers of apoptotic neurons expressing cleaved caspase‐3 after LCN2 treatment (n = 6). **P < .01, ***P < .001 compared with vehicle control (one‐way ANOVA and Newman–Keuls post hoc test). G, Confocal images showing the cleaved caspase‐3‐immunoreactive neurons (green) after treatment with recombinant human LCN2 protein (2 μg/mL). Nuclei were labeled with DAPI (blue). Scale bars, 25 μm

To assess the roles of LCN2 and its receptor, BOCT, in the survival of engrafted neurons, we determined the expression of LCN2 and BOCT in undifferentiated and differentiated LUHMES cells (Figure 9). *LCN2* mRNA was of low abundance both before and after differentiation (Figure 9A). Moreover, while the level of *BOCT* mRNA was low in undifferentiated LUHMES cells, it was induced 45‐fold after differentiation. Immunofluorescence staining revealed perinuclear expression pattern of BOCT in MAP2‐immunoreactive LUHMES neurons (Figure 9B), which is consistent with a recent study that reported expression of BOCT in dopaminergic neurons in the substantia nigra.^34^ To determine whether LCN2 directly mediated cell death of engrafted neurons, differentiated LUHMES neurons were treated with increasing concentrations of recombinant LCN2 protein. Addition of LCN2 protein reduced cell viability (Figure 9C) and increased apoptosis (Figure 9D‐G) of LUHMES neurons in a dose‐dependent manner.

## 4 DISCUSSION

**FIGURE 10:**
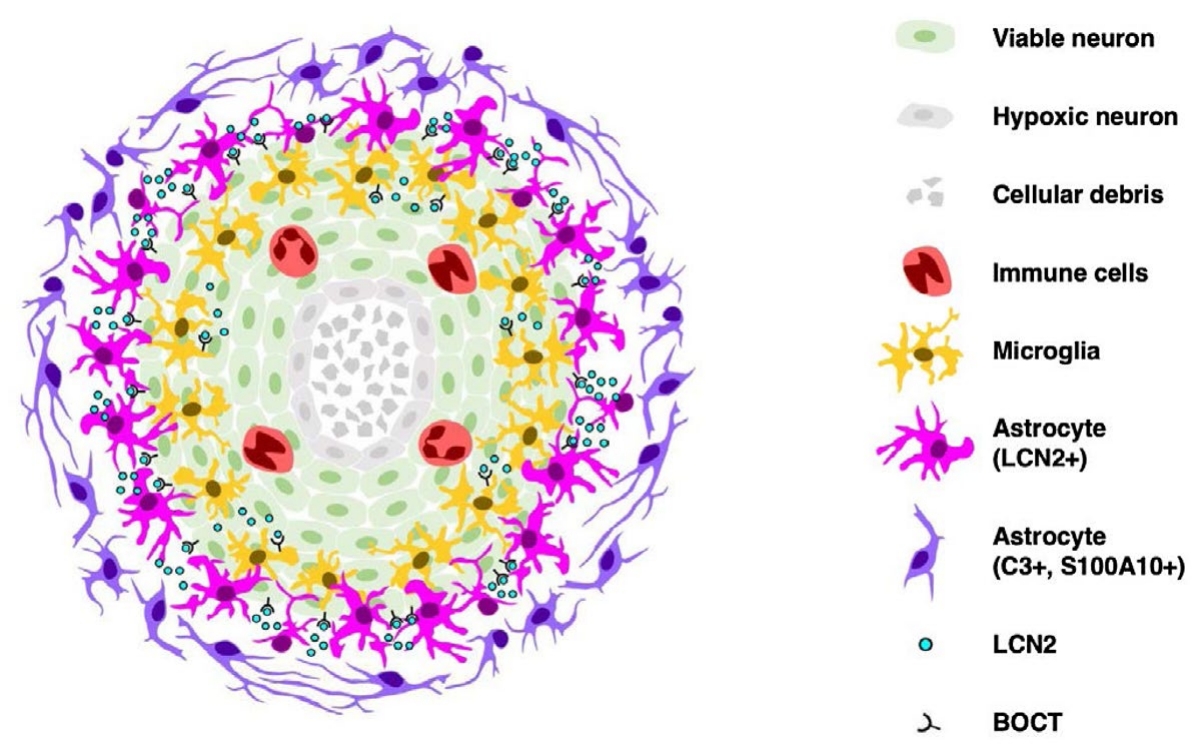
A proposed model depicting the role of LCN2 in neural transplantation. Transplantation of post‐mitotic neurons (green) in the brain recruits and activates resident microglia (yellow), peripheral immune cells (red), and astrocytes (purple and pink). Hypoxia and anoikis cause cell death in the core of engrafted neurons (grey). Activated astrocytes are broadly distributed around the graft site, while the activated microglia are recruited to the core of graft. LCN2 is induced in a subset of reactive astrocytes infiltrating in the engrafted site (pink). LCN2‐immunoreactive astrocytes express lower levels of A1 and A2 astrocytic markers (C3 and S100A10). LCN2 (cyan), released from reactive astrocytes and internalized by BOCT (black), induces apoptosis of engrafted neurons, possibly through intracellular iron sequestration

Cell replacement therapy has been considered as a potential therapeutic strategy for the treatment of neurological disorders.^3^ However, the host brain becomes hostile to engrafted cells due to robust inflammatory responses elicited after transplantation.^3^ Understanding the immune mechanisms involved in graft rejection is necessary to prevent the development of cytotoxic host brain environments and improve cell therapies for neurodegenerative disorders. We believe that this study is the first to demonstrate that LCN2 is a major mediator of graft rejection and neuroinflammation following neural transplantation (Figure 10). Our results provide clear evidence that expression of LCN2 is low‐to‐undetectable in normal brain tissues, and significantly upregulated in a subset of infiltrating astrocytes and neutrophils after transplantation. Recruitment of microglia, neutrophils, and monocytes was reduced after transplantation in LCN2 deficiency mice. Microglia were significantly polarized toward to the alternative M2 phenotypes after neural transplantation in LCN2 deficiency mice. Expression of *LCN2* and its receptor, *BOCT*, was low in undifferentiated LUHMES cells. Interestingly, only *BOCT*, but not its ligand, was expressed after differentiation in the dopaminergic‐like neurons. Rejection of engrafted neurons was attenuated in LCN2 deficiency mice as compared to wild‐type mice. Treatment with recombinant LCN2 protein directly induced apoptosis in the dopaminergic‐like neurons. These results support the hypothesis that astrocytic LCN2, internalized by BOCT, mediates cell death of engrafted neurons, and raises the intriguing possibility of using LCN2 inhibitors as an adjuvant treatment to improve the survival of engrafted neurons following cell replacement therapy.

In response to a variety of brain injuries, LCN2 protein is secreted extracellularly, where it can be pharmacologically targeted by therapeutic antibodies and inhibitors.^32^, ^33^ Developing neutralizing antibodies to reduce LCN2 neurotoxicity^33^ and small‐molecule inhibitors to block the expression and secretion of LCN2^35^ or interfere with the interaction between LCN2 and its receptors^19^ are potential therapeutic strategies.^32^ Considering that LCN2 is not expressed in the brain under normal conditions, one of the potential advantages of using LCN2 inhibitors in cell replacement therapy is the minimization of adverse side effects.

Intense activation of microglia and astrocytes after intracerebral transplantation has been strongly associated with graft rejection.^3^, ^36^ Activated astrocytes proliferate and migrate to the engrafted site, covering a large area surrounding the activated microglia and the graft (Figures 3, 4, 5, and 10). The spatial distribution of activated microglia and astrocytes, observed after transplantation, is in line with the findings reported in previous studies.^3^, ^8^, ^9^, ^10^, ^36^ Upregulation of LCN2 was detected in a subset of reactive astrocytes located centrally in the engrafted site, while C3‐ and S100A10‐immunoreactive astrocytes were broadly distributed around the graft (Figure 5). Reactive astrocytosis is a highly heterogeneous, dynamic, and context‐dependent process.^12^, ^16^, ^37^ Our findings using a model of neural transplantation revealed a unique spatial distribution of heterogeneous astrocytes around the graft. The expression of LCN2 vs C3, or LCN2 vs S100A10 in astrocytes was dependent on distance from the engrafted site.

LCN2‐immunoreactive astrocytes were in close contact with activated microglia after transplantation (Figure 3E). The expression of microglia marker Iba1 and markers for the alternative activation of microglia (Arg1, YM1, CCL22) was higher in the ipsilateral hemisphere of *LCN2* ^−/−^ mice 7 days after transplantation (Figure 7). Our results suggest that LCN2 is induced in reactive astrocytes and that secreted LCN2 mediates the M1/M2 polarization of adjacent microglia after transplantation.^37^ Previous studies have demonstrated that BOCT is expressed in microglia, and that LCN2 promotes M1 and suppresses M2 polarization of microglia in cell cultures and in LPS‐induced neuroinflammation models.^32^, ^38^ Our findings in this neural transplantation model support the roles of LCN2 in microglial polarization in response to brain injury.

Iron is an essential element for most organisms, and dysregulation of iron metabolism is highly associated with neurodegeneration and inflammation.^39^ Progressive degeneration of dopaminergic neurons in the substantia nigra and striatum constitutes the main pathological features of PD.^40^ Intracellular accumulation of iron is known to directly contribute to the pathogenesis of PD.^41^ LCN2 is an iron‐transporting protein that is internalized by its receptor BOCT, and then modulates the intracellular iron concentrations.^42^ LCN2 is upregulated in reactive astrocytes of the substantia nigra and striatum in mouse models of PD and in patients with PD.^34^ Pathogenic upregulation of LCN2 contributes to increased cellular uptake of iron and neurotoxicity, resulting in apoptosis of dopaminergic neurons. Our results, using differentiated LUHMES cells (Figure 9), also showed LCN2‐mediated neurotoxicity in dopaminergic neurons. Iron dysregulation has been associated with higher rates of graft rejection and inferior outcomes after kidney,^43^ liver,^44^ lung,^45^ and heart^46^ transplantation in patients, and after heart transplantation in mice.^47^ Association between iron dysregulation and neural transplantation has, however, not yet been established. Our results indicate links connecting iron, LCN2, and neural transplantation.

## 5 CONCLUSIONS

This study provides a plausible mechanism by which LCN2, likely secreted from reactive astrocytes and internalized by BOCT, mediates changes in intracellular iron concentrations in engrafted neurons and causes subsequent neuronal cell death after transplantation. Our results indicate an important role for astrocytic LCN2 in graft rejection and strongly suggest that LCN2 inhibitors could prove useful in cell replacement therapy.

## CONFLICT OF INTEREST

The authors have declared that no competing interests exist.

## AUTHOR CONTRIBUTIONS

All authors had full access to all the data in the study and take responsibility for the integrity of the data and the accuracy of the data analysis. Study concept and design: W.‐H. Chou. Acquisition of the data: Y.‐C. Weng, Y.‐T. Huang, I.‐C. Chiang. Analysis and interpretation of the data: Y.‐C. Weng, Y.‐T. Huang, I.‐C. Chiang, P.‐J. Tsai, Y.‐W. Su, W.‐H. Chou. Drafting of the manuscript: W.‐H. Chou, Y.‐C. Weng, Y.‐W. Su. Critical revision of the article for important intellectual content: W.‐H. Chou, Y.‐C. Weng, Y.‐W. Su. Statistical analysis: W.‐H. Chou, Y.‐C. Weng, Y.‐T. Huang, I.‐C. Chiang. Obtained funding: W.‐H. Chou. Study supervision: W.‐H. Chou.

## Supporting information

Fig S1

Fig S2

Fig S3

Fig S4

Fig S5

Table S1

Text S1
