# Supplementary figures and images for: Lipocalin‐2 mediates the rejection of neural transplants
Source: FASEB J. 2021 Jan 9;35(2):e21317. doi: 10.1096/fj.202001018R (PMC12315500; doi:10.1096/fj.202001018R)

Suppl Fig 1

(A)

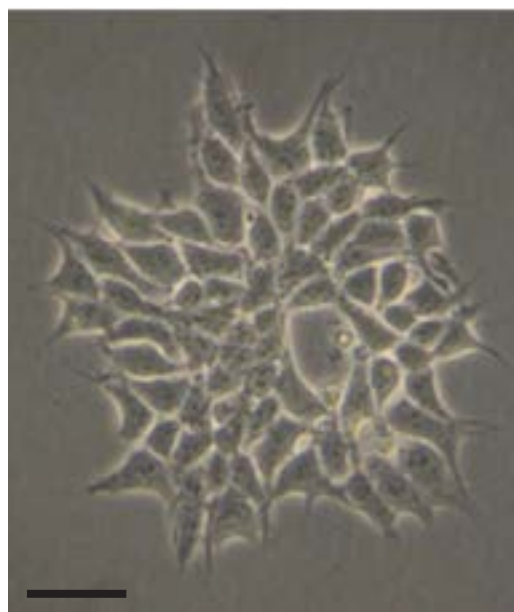

(B)

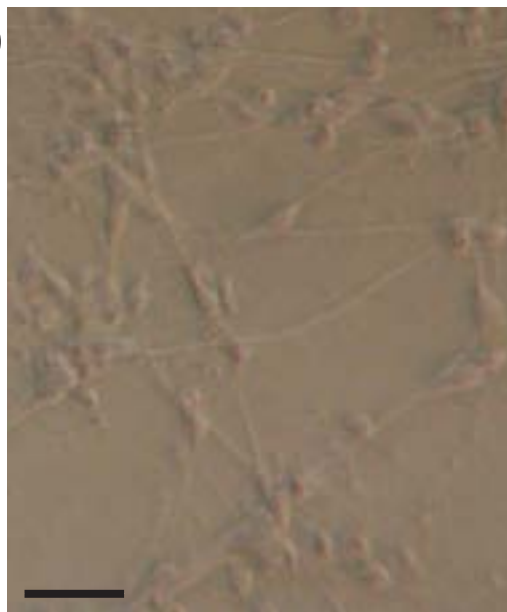

(C)

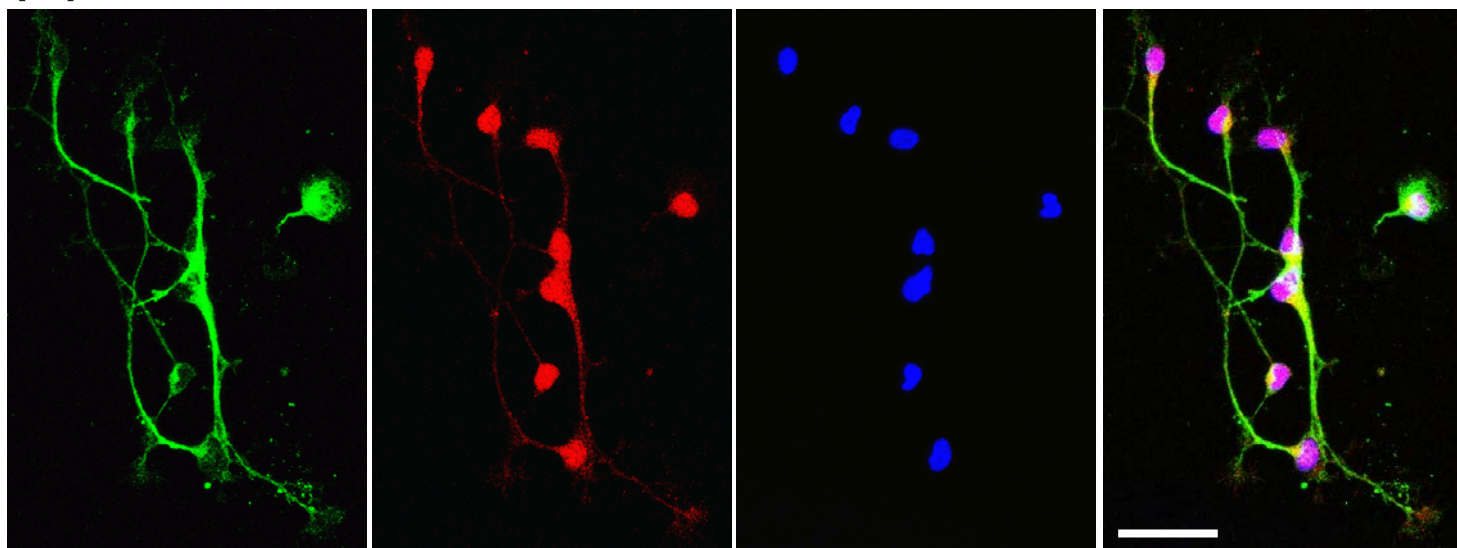

Supplement: Supplementary file 1 — Fig S1 [file FSB2-35-e21317-s007.pdf]

Suppl Fig 2

(A)

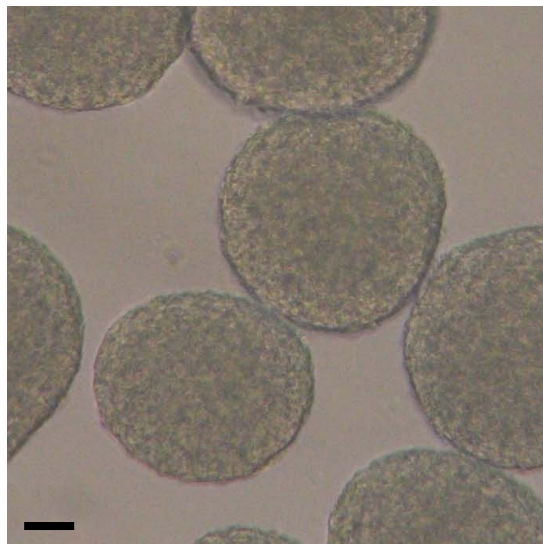

(B)

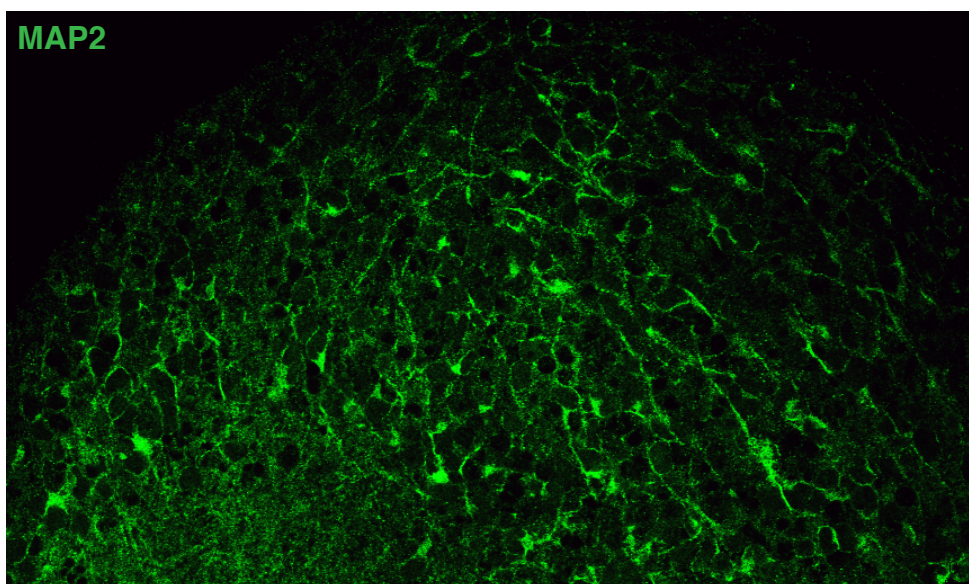

(C)

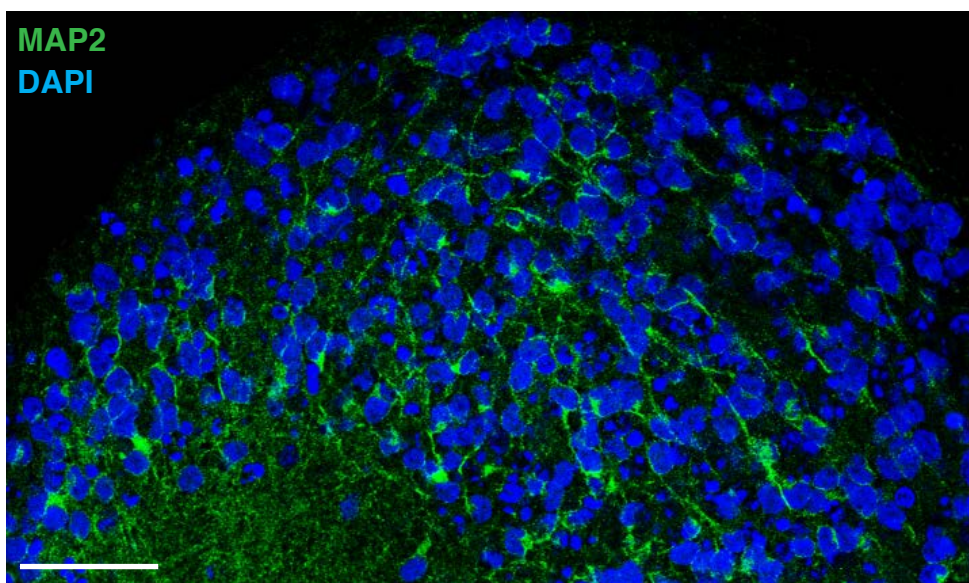

Supplement: Supplementary file 2 — Fig S2 [file FSB2-35-e21317-s006.pdf]

Suppl Fig 3

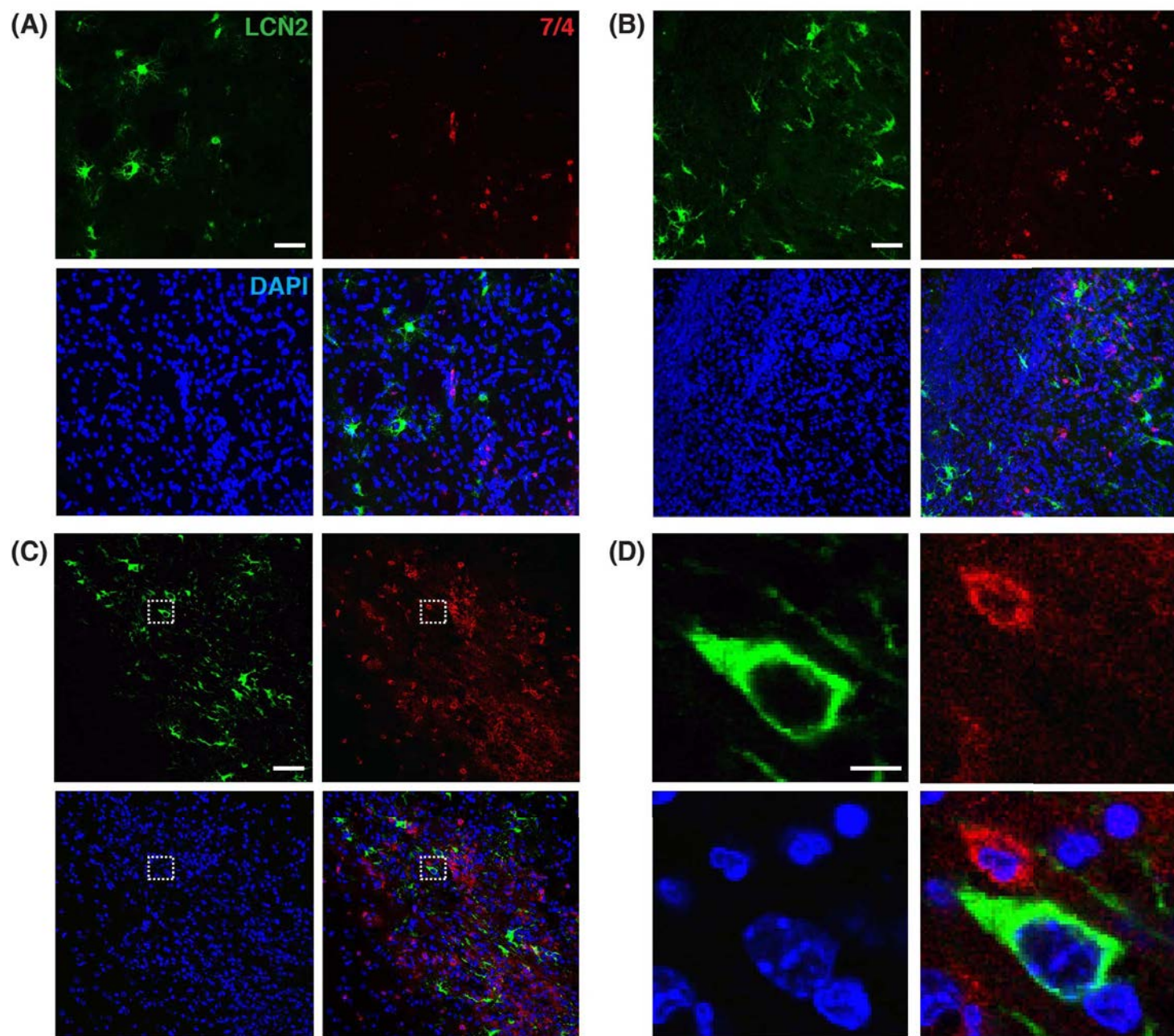

Supplement: Supplementary file 3 — Fig S3 [file FSB2-35-e21317-s005.pdf]

Suppl Fig 4

(A)

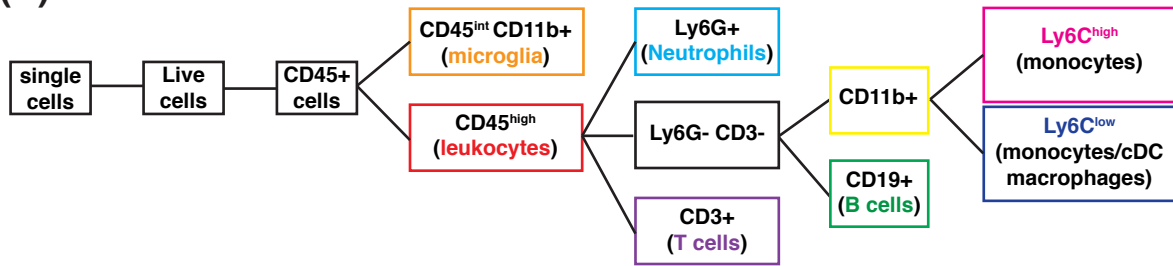

(B)

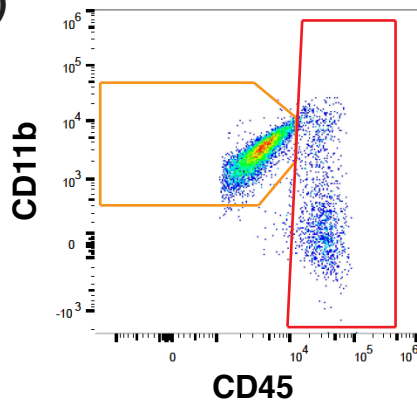

(C)

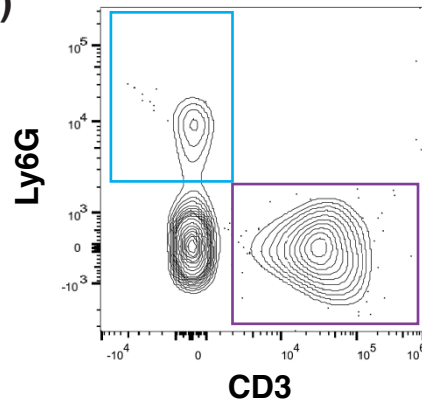

(D)

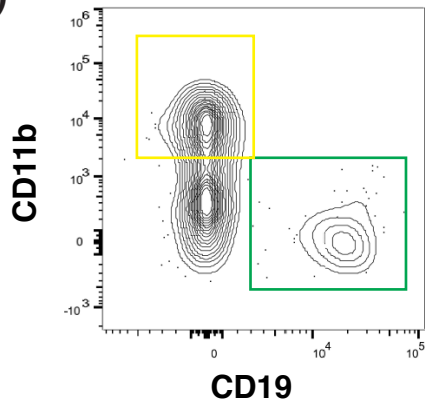

(E)

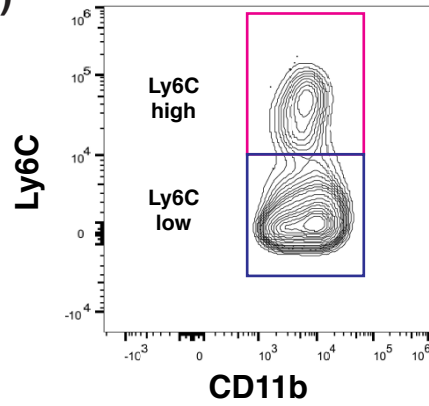

Supplement: Supplementary file 4 — Fig S4 [file FSB2-35-e21317-s001.pdf]

## Suppl Fig 5

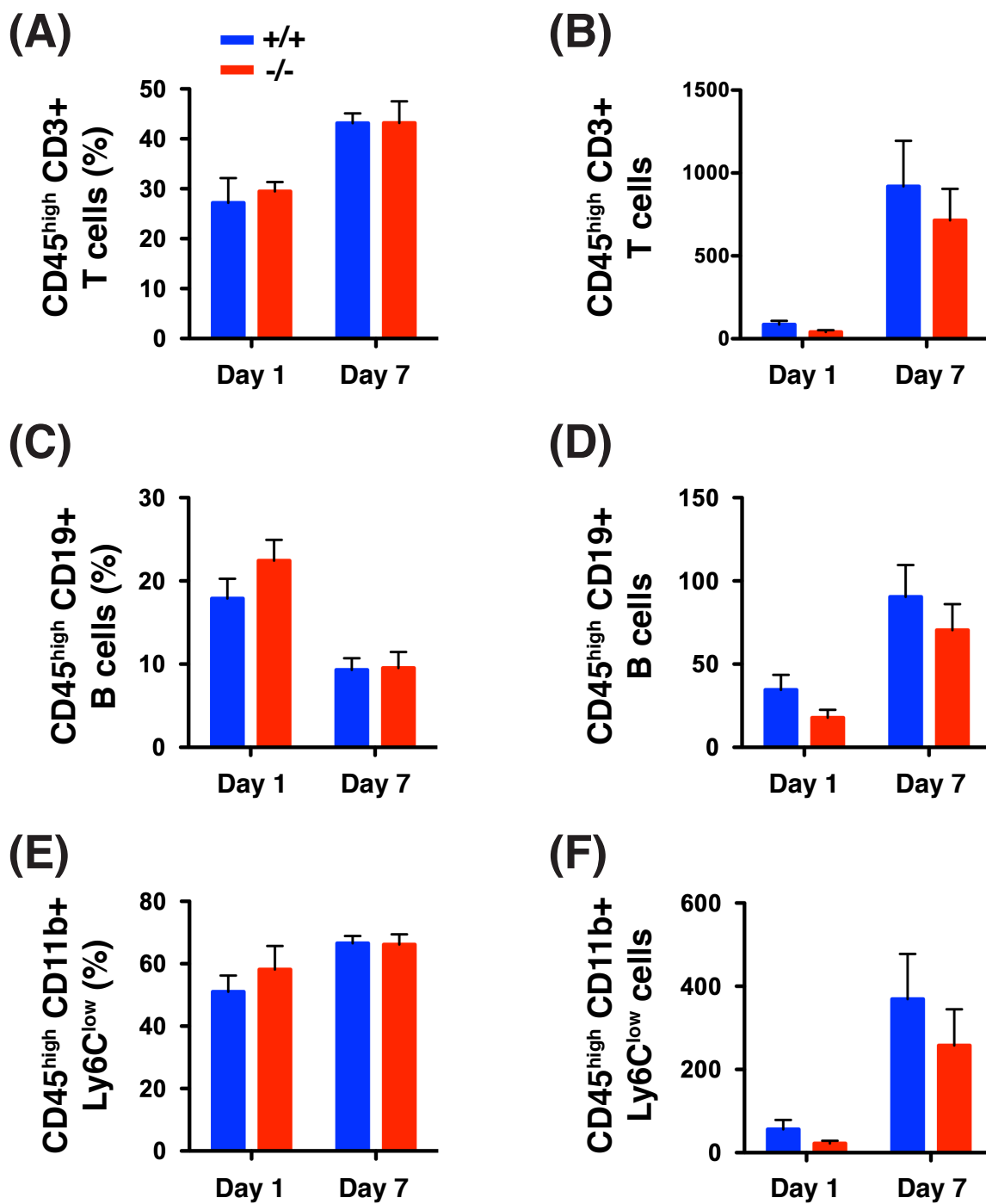

Supplement: Supplementary file 5 — Fig S5 [file FSB2-35-e21317-s002.pdf]
