# Supplementary material for: Lipocalin‐2 mediates the rejection of neural transplants
Source: FASEB J. 2021 Jan 9;35(2):e21317. doi: 10.1096/fj.202001018R (PMC12315500; doi:10.1096/fj.202001018R)
Supplement: Supplementary file 6 — Table S1 [file FSB2-35-e21317-s003.docx]

**Supplemental Table 1.** Statistics for the pairwise comparisons.

|  | *F* statistics | *df* | Overall *P* value |
| --- | --- | --- | --- |
| Figure 2B | 12.12 | 23 | < 0.0001 |
| Figure 3F | 3.424 | 4 | 0.0024 |
| Figure 4G | 1.338 | 4 | 0.8554 |
| Figure 5I | 46.5 | 8 | 0.0002 |
| Figure 5J | 0.3362 | 8 | 0.7271 |
| Figure 5K | 1.643 | 58 | < 0.0001 |
| Figure 5L | 2.770 | 58 | 0.002 |
| Figure 6A – Day 1 | 1.311 | 8 | 0.7993 |
| Figure 6A – Day 7 | 7.350 | 8 | 0.0792 |
| Figure 6B – Day 1 | 161.8 | 8 | 0.0002 |
| Figure 6B – Day 7 | 5.503 | 8 | 0.1274 |
| Figure 6C – Day 1 | 9.156 | 8 | 0.0543 |
| Figure 6C – Day 7 | 3.312 | 8 | 0.2728 |
| Figure 6D – Day 1 | 27.09 | 8 | 0.0074 |
| Figure 6D – Day 7 | 1.080 | 8 | 0.9422 |
| Figure 6E – Day 1 | 2.127 | 8 | 0.4827 |
| Figure 6E – Day 7 | 1.990 | 8 | 0.5216 |
| Figure 6F – Day 1 | 13.58 | 8 | 0.0269 |
| Figure 6F – Day 7 | 3.622 | 8 | 0.2404 |
| Figure 7A | 20.97 | 23 | < 0.0001 |
| Figure 7B | 8.994 | 23 | 0.0002 |
| Figure 7C | 0.4309 | 23 | 0.8211 |
| Figure 7D | 9.848 | 23 | 0.0001 |
| Figure 7E | 6.671 | 23 | 0.0011 |
| Figure 7F | 4.261 | 23 | 0.0099 |
| Figure 7G | 36.80 | 23 | < 0.0001 |
| Figure 7H | 7.745 | 23 | 0.0005 |
| Figure 7I | 9.703 | 23 | 0.0001 |
| Figure 8B | 631.6 | 8 | < 0.0001 |
| Figure 9A – LCN2 | 4.303 | 8 | 0.0005 |
| Figure 9A – BOCT | 197.7 | 8 | < 0.0001 |
| Figure 9C | 20.02 | 19 | < 0.0001 |
| Figure 9D | 28.84 | 23 | < 0.0001 |
| Figure 9F | 21.45 | 20 | < 0.0001 |
| Suppl Fig 5A – Day 1 | 7.091 | 8 | 0.0841 |
| Suppl Fig 5A – Day 7 | 4.876 | 8 | 0.1541 |
| Suppl Fig 5B – Day 1 | 3.687 | 8 | 0.2343 |
| Suppl Fig 5B – Day 7 | 2.081 | 8 | 0.4954 |
| Suppl Fig 5C – Day 1 | 1.126 | 8 | 0.9112 |
| Suppl Fig 5C – Day 7 | 1.931 | 8 | 0.5395 |
| Suppl Fig 5D – Day 1 | 3.568 | 8 | 0.2455 |
| Suppl Fig 5D – Day 7 | 1.479 | 8 | 0.7138 |
| Suppl Fig 5E – Day 1 | 2.127 | 8 | 0.4827 |
| Suppl Fig 5E – Day 7 | 1.990 | 8 | 0.5216 |
| Suppl Fig 5F – Day 1 | 11.98 | 8 | 0.0338 |
| Suppl Fig 5F – Day 7 | 1.555 | 8 | 0.6793 |
