# Supplementary material for: Lipocalin‐2 mediates the rejection of neural transplants
Source: FASEB J. 2021 Jan 9;35(2):e21317. doi: 10.1096/fj.202001018R (PMC12315500; doi:10.1096/fj.202001018R)
Supplement: Supplementary file 7 — Text S1 [file FSB2-35-e21317-s004.docx]

**Supplemental Data legends**

**Supplemental Figure 1** Differentiation of 2D LUHMES cells. Brightfield images of 2D LUHMES cells in monolayer that were collected before (A), and 6 days after (B), differentiation. C, Confocal images showing the expression of dopaminergic neuron markers in LUHMES cells 6 days after differentiation. Neurons were immunostained with antibodies recognizing MAP2 (green) and TH (red). Nuclei were labeled with DAPI (blue). Scale bars, 50 μm.

**Supplemental Figure 2** Differentiation of 3D LUHMES cells. A, Brightfield image of 3D LUHMES neurospheres, in aggregate formation, collected 4 days after differentiation. B and C, Confocal images showing the expression of the neuronal marker MAP2 (green) in a LUHMES neurosphere 4 days after differentiation. Nuclei were labeled with DAPI (blue). Scale bars, 50 μm.

**Supplemental Figure 3** Immunoreactivity of LCN2 (green) and 7/4 (red) in the mouse striatum at day 3 (A), day 5 (B), and day 7 (C) after transplantation. D, Enlarged views of the boxed areas in C showing that LCN2 (green) is not expressed in 7/4-immunoreactive neutrophils (red) at day 7 after transplantation. Nuclei were labeled with DAPI (blue). Scale bars, 50 μm in A–C and 5 μm in D.

**Supplemental Figure 4** (A) Schematic diagram showing the gating strategy for flow cytometry. Relative distribution of CD45^int^ CD11b+ microglia (orange outlined) and CD45^high^ leukocytes (red outlined) (B), CD45^high^ Ly6G+ neutrophils (cyan outlined) and CD45^high^ CD3+ T cells (purple outlined) (C), CD45^high^ CD11b+ cells (yellow) and CD45^high^ CD19+ B cells (green outlined) (D), CD45^high^ CD11b+ CD45^high^ monocytes (pink outlined) and CD45^high^ CD11b+ CD45^low^ monocytes/cDC/macrophages (blue outlined) (E) in the ipsilateral hemisphere at day 7 after transplantation. cDC, classical dendritic cells; FSC, forward scatter.

**Supplemental Figure 5** Recruitment of immune cells after neural transplantation. Flow cytometric analysis of the percentages (A, C, E) and numbers (B, D, F) of CD45^high^ CD3+ T cells (A, B), CD45^high^ CD19+ B cells (C, D), and CD45^high^ CD11b+ Ly6C^low^ monocytes/cDC/macrophages (E, F) in the ipsilateral hemisphere at day 1 and day 7 after transplantation (*n* = 5 per group). The percentages and numbers of immune cells were compared between *LCN2^+/+^* and *LCN2^-/-^* mice at day 1 or day 7 after transplantation using the two-tailed, unpaired *t*-test; no statistically significant differences were detected.
